# Self-Healing Concrete Using Rubber Particles to Immobilize Bacterial Spores

**DOI:** 10.3390/ma12142313

**Published:** 2019-07-19

**Authors:** Hongyin Xu, Jijian Lian, Maomao Gao, Dengfeng Fu, Yue Yan

**Affiliations:** State Key Laboratory of Hydraulic Engineering Simulation and Safety, Tianjin University, Tianjin 300350, China

**Keywords:** self-healing concrete, rubber particles, cracks, strength

## Abstract

Bacteria-based self-healing concrete is a construction material used to repair cracks in concrete, in which the bacterial spores are immobilized by bacteria carriers. However, the currently available bacteria carriers are not always suitable due to a complicated procedure or high cost. To develop a more suitable bacteria carrier as well as improve the anti-crack capability of self-healing concrete, in this study we evaluate the feasibility of using rubber particles as a novel bacteria carrier in self-healing concrete. Two types of self-healing concrete are prepared with rubber particles of different sizes to quantify the crack-healing effect. In addition, the fluidity and mechanical properties of the self-healing rubber concrete are compared with those of plain concrete and normal rubber concrete. The experimental results show that the self-healing rubber concrete with a particle size of 1~3 mm has a better healing capacity than the self-healing rubber concrete with a particle size of 0.2~0.4 mm, and the width value of the completely healed crack is 0.86 mm. The self-healing rubber concrete has a higher slump than the plain concrete and normal rubber concrete. According to the strength tests, the compressive strengths of the self-healing rubber concrete are low early on but they exceed those of the corresponding normal rubber concrete at 28 days. Moreover, the self-healing rubber concrete has higher splitting tensile strengths than the plain concrete and a better anti-crack capability. The results of a comparison to the other two representative bacterial carriers indicate that rubber particles have potential to be a widely used bacteria carrier for practical engineering applications in self-healing concrete.

## 1. Introduction

Concrete is a widely used construction material as it has a high compressive strength and good durability. However, a major concern with concrete is that it is vulnerable to cracking under the influence of temperature fluctuations and external load due to its low tensile strength and poor deformation ability [1,2]. The cracks in concrete provide a path for water and other aggressive substances, such as sulfate and chloride ions, to penetrate into the concrete matrix, resulting in steel reinforcement corrosion, strength reduction, and structural failure [3,4,5]. Various methods have been developed to heal the cracks in concrete. They can be divided into two categories: man-made repairs and self-healing repairs. Man-made methods apply repairing agents from outside the cracks to fill them after they are detected, and are mainly used to repair large cracks and difficult to repair small and deep cracks [5,6]. Self-healing approaches pre-bury the repairing agents in the concrete matrix and can repair cracks automatically once they appear, which can save on operation costs compared to the man-made methods [7]. According to previous studies [8,9,10,11], the self-healing approaches include autogenous, adhesive-based, mineral-admixtures-based, and bacteria-based methods. The bacteria-based self-healing repair method has attracted much attention due to its better healing capacity and environmentally friendly character.

The bacteria-based self-healing of concrete cracks is based on Microbial Induced Calcite Precipitate (MICP), which involves the application of mineral-producing bacteria to precipitate calcium carbonate onto the crack face in the concrete [12,13]. The bacterial spores and other repairing agents are mixed into the fresh concrete when manufacturing self-healing concrete. They remain dormant in the concrete matrix and have the potential to germinate before cracks appear. When the concrete cracks, the bacteria begin to proliferate after activation by the ingress of water and oxygen via the cracks and produce calcium carbonate on the crack faces. Thus, the generated crystals can block the cracks and result in the self-repair of the cracks [1,6].

Due to the harsh environment inside the concrete, the bacterial spores must be protected from squeezing and high alkalinity by a bacteria carrier to ensure that the bacteria have space to grow and a mineralized environment [14]. A range of bacteria carriers have been adopted in previous studies and they can be divided into two categories depending on the immobilization mode: encapsulated and adsorbed (see Table 1). The encapsulated mode injects bacterial spore suspension and other repair agents into microcapsules made of special materials, such as glass [6], poly [15], and melamine [16]. These microcapsules are embedded in the concrete matrix and rupture as soon as cracks appear. Thus, the healing agent is released onto the crack face and crystals are produced to heal the cracks [17,18]. Self-healing by the encapsulation method can provide high-quality healing in terms of being able to repair cracks with a wider range of widths [2,8]. However, the encapsulated method always involves a complicated procedure and professional equipment is required to produce microcapsules, resulting in a relatively high cost. In the adsorbed method, bacterial spores are immobilized by porous materials with high porosity and a high capacity for water absorption. Their highly porous structure can provide sufficient oxygen, water, and growth space to bacteria after the concrete cracks. Various attempts have been made to develop porous bacteria carriers and the results vary across different studies [1,7,19,20]. To date, an ideal bacteria carrier has not been obtained. Thus, it is necessary to develop more carrier techniques for bacteria-based self-healing concrete.

Scrap rubber tires are a major form of environmental solid waste that are dangerous not only due to being a potential environmental threat, but also because they constitute a fire hazard and provide a breeding ground for rats, mice, and mosquitoes [21,22]. One possible use for scrap tires is to crush them into rubber particles and then incorporate them into concrete to decrease the brittle behavior of the concrete. This results in a lower crack width in rubber concrete than in plain concrete [22,23]. Moreover, the rubber particles have the characteristics of surface roughness and high porosity, which make them an attractive bacteria-carrier candidate. In this study, we investigate the feasibility of using rubber particles to immobilize bacterial spores in self-healing concrete and to improve the anti-crack capability of self-repairing concrete. Two types of self-healing concrete specimens with different rubber particle sizes are prepared to quantify the crack-healing effect. The slump and the mechanical properties of the self-healing rubber concrete are compared to those of plain concrete and normal rubber concrete. In addition, a comparison of rubber particles to other bacteria carriers is performed.

## 2. Materials and Methods

### 2.1. Materials

**Figure 1:**
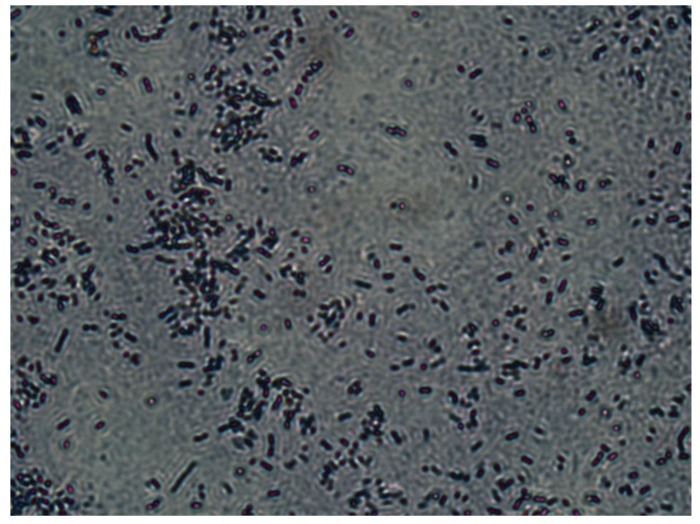
A micrograph of bacterial spores.

A ureolytic-type bacterium, Sporosarcina pasteurii ATCC11859, was used in this study. It was cultured in a sterilized liquid medium containing 20 g/L of yeast extract, 10 g/L of ammonium sulfate, and 17.5 g/L of Tris regent. After cultivation in a shaker for 24 h at a temperature of 30 °C and a rotational speed of 220 rpm, the bacteria reached a stable growth period. Then, the bacterial suspension was centrifuged at 4000 rpm for 30 min to separate the bacterial cells from the cultivation solution. The supernatant was dispensed with and the remaining bacterial cells were re-suspended in 9 g/L of NaCl solution. The re-suspended bacterial solution was placed in an oven at 30 °C for 14 days, during which time the bacteria remained in starvation. After that, almost all bacterial cells turned into bacterial spores. This was confirmed by microscopic observation of the re-suspension solution as shown in Figure 1, in which the bacterial spores are dyed red. The concentration of spores in the bacterial spore suspension was determined to be 2.5E9 spores/mL by a plate count method [24]. The bacterial spore suspension was stored at 4 °C prior to the tests.

**Figure 2:**
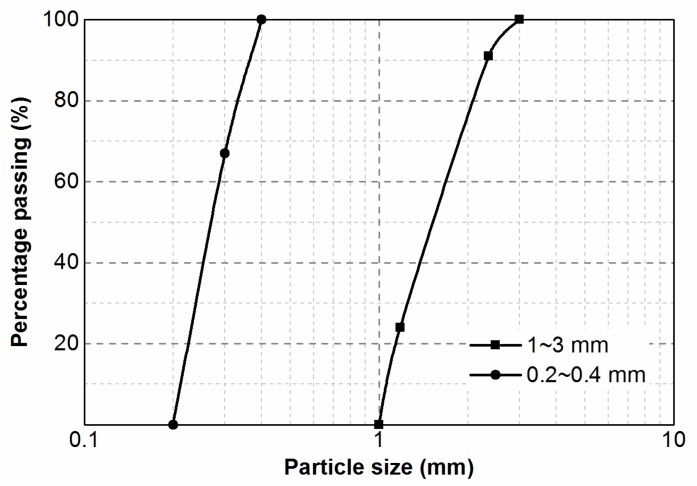
Particle size distributions of the rubber particles.

Concrete specimens were prepared with ordinary Portland cement (P.O. 42.5, Tianjin, China), tap water, local natural sand (distributed within 0~5 mm), and coarse aggregate (gravel, distributed within 5~25 mm). We adopted two rubber particle sizes (1~3 mm and 0.2~0.4 mm) in this study, whose particles size distributions were displayed in Figure 2. All of the rubber particles were obtained by cutting scrap rubber tire casings, which contain 37% natural rubber, 25% butadiene rubber, 30% carbon black, and 8% other additives. All other chemicals used in this study were of Analytical Reagent (AR) grade.

### 2.2. Concrete Specimen Preparation

**Figure 3:**
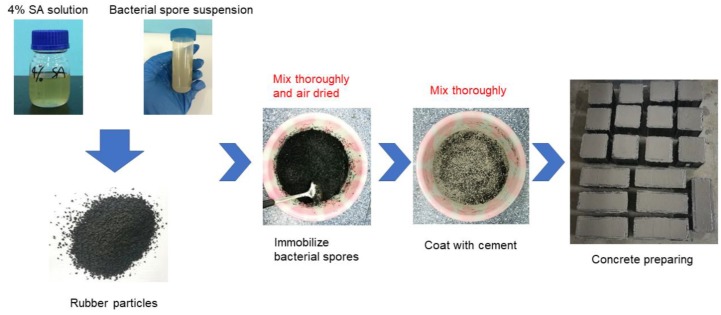
Preparation of self-healing concrete specimens via immobilization of bacterial spores in rubber particles.

Figure 3 shows the process for preparing the self-healing concrete specimens by immobilizing bacterial spores in rubber particles. The details of the procedure are as follows:

1. The bacterial spore suspension was prepared according to the method described in Section 2.1. Then, the rubber particles were mixed with the bacterial spore suspension and 4% sodium alginate (SA) solution. The ratio of SA solution, bacterial spore suspension, and rubber particles was 1 mL:1 mL:2.2 g. The role of the SA solution was to adhere the bacterial spores to the rubber particles and coat the rubber particles to protect the spores from leaving the pores in the rubber particles. The proportion of these three substances resulted from ensuring that there were 3.75E13 bacterial spores per m^3^ of concrete and that there was sufficient SA solution to disperse the spores under the premise that the amount of rubber particles could be determined.
2. The mixture of the three substances was stirred and then dried in a cool and ventilated place until the SA had completely solidified.
3. Before preparing the self-healing concrete, the spore-containing rubber particles were coated with cement (1 kg of rubber particles with 2 kg of cement, note that this cement was counted in the mixing proportion of the concrete) by a stirring method. Thus, the cement can protect SA from directly contacting with free water and slow down the dissolution of SA in the fresh concrete.
4. When manufacturing the self-healing concrete specimens, the nutrients for bacterial growth (yeast extract and ammonium sulfate) and the necessities for bacterial mineralization (calcium acetate and urea) were mixed into the tap water, which was added to the concrete mixture following the coarse aggregate, cement, rubber particles and sand.

Five types of concrete were prepared in this study: two types of self-healing rubber concrete, each with a different rubber particle size (1~3 mm (SRC-L) and 0.2~0.4 mm (SRC-S)), plain concrete (C), and two types of normal rubber concrete (RC-L and RC-S). The latter three types of concrete were used as contrasts of the self-healing concretes’ properties. The water-to-cement ratio of these mixtures was 0.54. Note that the rubber particles replaced 10% of the volume of the sand in the normal rubber concretes and the self-healing rubber concretes to effectively decrease the brittleness of the concrete and not reduce the strength of the concrete too much at the same time [25]. The mixing proportions of the specimens are shown in Table 2. All specimens were cured under the conditions of 20 ± 2 °C and higher than 95% humidity for different time periods.

### 2.3. Bacterial Growth Test with SA and Rubber Particles

To determine the effects of rubber particles and SA on bacterial spore growth, six bottles of 100 mL of fresh liquid medium were prepared then separately inoculated with: 1 mL of bacterial spore suspension (I); 1 mL of bacterial spore suspension and 1 mL of SA solution (II); 1 mL of bacterial spore suspension and 2.2 g of sterilized rubber particles (III); 1 mL of bacterial spore suspension and 2.2 g of unsterilized rubber particles (IV); 2.2 g of sterilized spore-containing rubber particles (V); and 2.2 g of unsterilized spore-containing rubber particles (VI). All media were cultured in a shaker at a temperature of 30 °C and a rotational speed of 220 rpm for four days. The urease activities in the media were detected using a conductivity method [26] to estimate the amount of bacterial growth.

### 2.4. Crack Manufacturing Methods and Self-Healing Incubation Conditions

**Figure 4:**
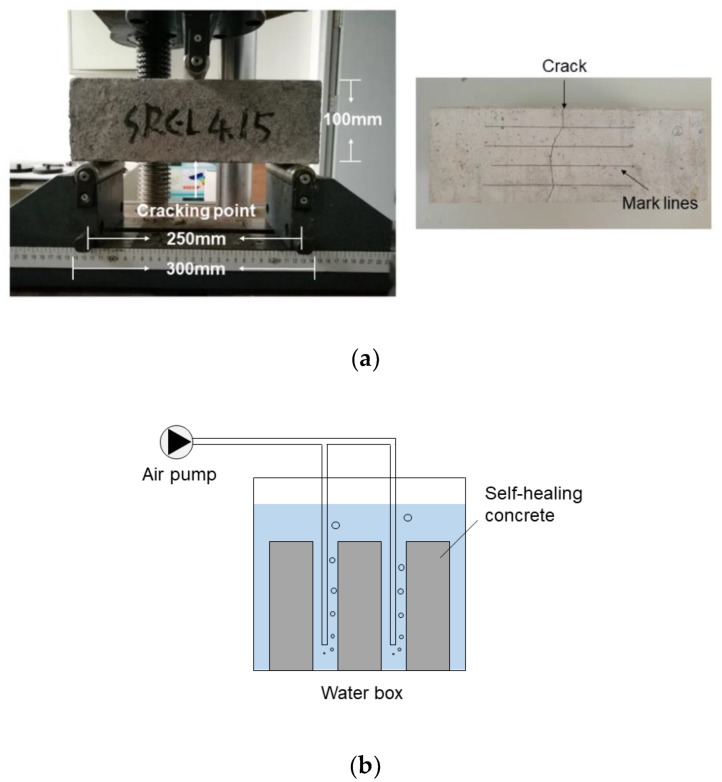
The creation of cracks (**a**) and the incubation conditions for the self-healing concrete (**b**).

Three prismatic specimens (100 mm × 100 mm × 300 mm) were prepared to observe the capacity of each type of concrete to self-heal cracks. The cracks were created at the concrete age of 7 days using a three-point bending method (see Figure 4a). The specimens were loaded at a rate of 0.5 mm/min until a clear crack appeared at the subface. The three specimens of each type of concrete were loaded to a different extent to obtain cracks with different widths distributed in the range 0.2~0.9 mm. Four mark lines were drawn on the cracked surface of the concrete and the intersections of the mark lines and the cracks were used to monitor the progression of crack self-healing, as shown in Figure 4a.

After the cracks were created, the concrete specimens were placed in a water box for crack healing at a temperature of 20 °C, as shown in Figure 4b. An air pump was used to provide the oxygen that the concrete needed to self-heal. The self-healing incubation period lasted for 28 days, and the results of crack repair were observed at 3 days, 7 days, 14 days, and 28 days.

### 2.5. Concrete Properties Testing Methods

The slump of fresh concrete, the compressive strength of concrete, and the splitting tensile strength of concrete were investigated according to the Code for Hydraulic Concrete in China (SL 352-2006).

### 2.6. Compressive Strength Recovery Test

Three cubic specimens (150 mm × 150 mm × 150 mm) of each type of concrete were pre-compressed to their maximum strengths at the age of 7 days. These specimens with many cracks were placed under the self-healing incubation conditions for 21 days. Afterward, their compressive strengths were determined again. The strength regain of each type of concrete is expressed as the average of three specimens, which was calculated by Equation (1).

Strength regain (%) = *F*/*F*_0_ × 100 (1)

where *F* and *F*_0_ are the compressive strength at the age of 28 days of the specimens with and without precompression, respectively, in MPa.

## 3. Results

### 3.1. Bacterial Growth under the Effect of SA and Rubber Particles

**Figure 5:**
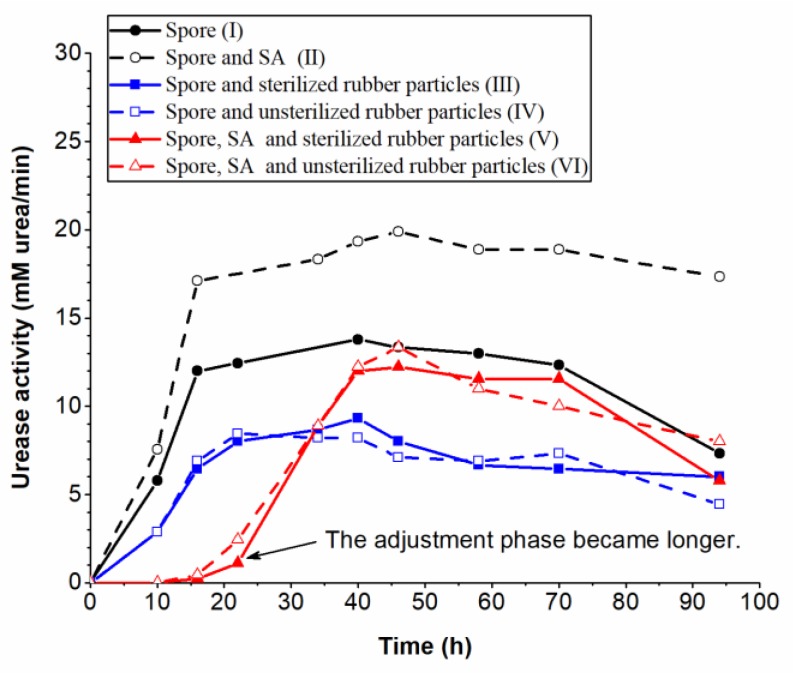
Bacterial growth under the effect of sodium alginate (SA) and rubber particles.

Figure 5 displays the urease activities in the bacterial growth process under the effect of SA and rubber particles. The results show that the bacterial spores germinated and proliferated in all six tests, producing urease in the medium. Test (I) was the control test, whose urease activity began to increase rapidly (logarithmic phase) after a short adjustment phase, until a urease activity of about 12 mM urea/min was reached at 15 h. Then, the urease activity was maintained during a stable phase before it declined at about 70 h (the decline phase). Tests (II), (III), and (IV) were similar to the control test, reaching the stable phase at about 15 h. However, the urease activities in the stable phase in Test (II) were about 19 mM urea/min, 58% higher than those in the control test, and about 8 mM urea/min in Tests (III) and (IV), 33% lower than those in the control test. This indicates that SA was favorable for the urease activities and the rubber particles were adverse. In Tests (V) and (VI), the urease activities did not increase until 15 h after inoculation, implying that the adjustment phase was longer in these two tests. This may be because it took a long time for the bacteria to adjust to the new substances in the environment. In addition, the bacterial spores in Tests (V) and (VI) were released slowly into the medium because of the coat of SA, resulting in a lag in the increase of bacterial concentration in the medium. At about 40 h, the urease activities in Tests (V) and (VI) reached the maximum value of about 12 mM urea/min, which was similar to that in the control test, suggesting that the bacterial spore immobilization method is feasible and does not affect the bacterial activities in the stable phase. Comparing Test (III) with Test (IV), as well as Test (V) with Test (VI), it can be found that the sterilization of rubber particles had no effect on the bacterial activities. This proves the feasibility of this bacterial spore immobilization method for large-scale engineering applications.

### 3.2. Optical Determination of Crack-Healing Capacity

**Figure 6:**
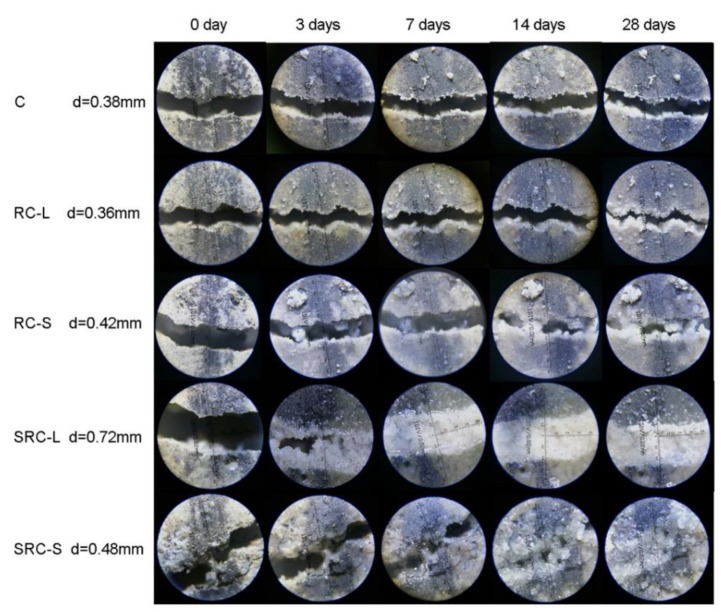
The results of the optical determination of the crack-healing capacity.

Figure 6 displays the microscopic images of representative cracks of the five types of specimens at different healing times. Some white healing compound can be observed visibly in the cracks of all of the specimens, implying that these five types of concrete all had some self-healing capacity. However, this capacity was relatively limited in the C, RC-L, and RC-S specimens. After 28 days of healing, the cracks in these three types of concrete were only partially healed, with an initial crack width of less than 0.45 mm. In the SRC-L and SRC-S specimens, the wider cracks (0.72 mm and 0.48 mm, respectively) were completely repaired.

**Figure 7:**
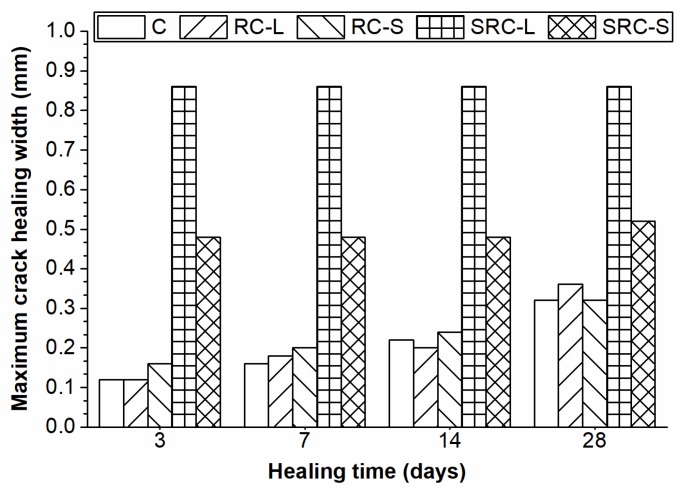
Maximum healed crack width at different healing times.

Figure 7 illustrates the maximum values of the widths of completely healed cracks for the five types of concrete at different healing times. As the healing time increased, the specimens with no self-healing agent (C, RC-L, and RC-S) exhibited improving values of maximum healed crack width, but only reached a value of 0.25 mm at 28 days. In contrast, the self-healing rubber concretes (SRC-L and SRC-S) showed a much better healing capacity, especially the SRC-L specimens that had a maximum healed crack width of 0.86 mm at three days. The reason behind this phenomenon was that the specimens with no self-healing agent were healed only by the hydration of unhydrated cement particles [7], but the self-healing rubber concretes were also healed by the bacterial mineralization. Therefore, the self-healing technique using rubber particles to immobilize bacterial spores was effective and greatly improved the self-healing capacity of the concrete.

To quantify the crack-healing capacity, 10 measurement points were selected and averaged along with the length of each crack in the microscopic image. The crack widths at the measurement points were measured. The crack healing percentage was determined as follows:

Healing percentage (%) = (*d*_0_ − *d*t)/*d*_0_ × 100 (2)

where *d*_0_ is the average initial crack width and *d*_t_ is the average width measured at the healing time of all 10 measurement points.

**Figure 8:**
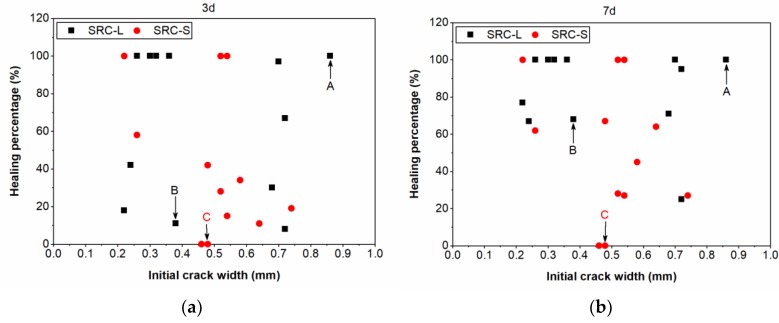

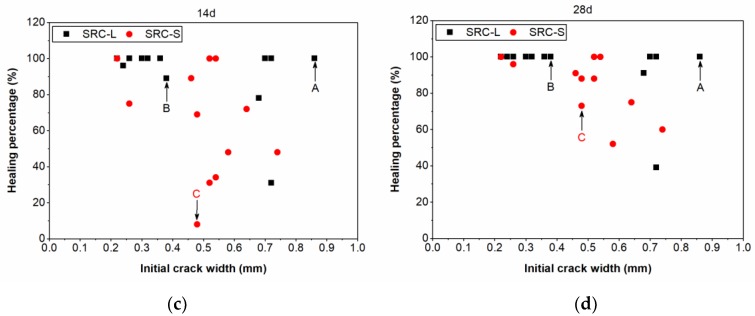
Crack healing percentage against the initial crack width at different healing times: three days (**a**), seven days (**b**), 14 days (**c**), and 28 days (**d**).

Figure 8 compares the crack healing percentage of the SRC-L concrete with that of the SRC-S concrete at different healing times. At the healing time of three days (Figure 8a), half of the cracks in the SRC-L concrete specimens had been completely repaired from an initial width of 0.22~0.86 mm, and only three cracks with an initial crack width of 0.22~0.54 mm in the SRC-S concrete had the healing percentage of 100%. In addition, we found that the initial crack width had no influence on the healing percentage. For example, Crack A in the SRC-L concrete with a width of 0.86 mm was completely healed, but Crack B with a width of 0.38 mm had a healing percentage of 11%. The widest crack had the best healing capacity, which may have resulted from the higher bacterial concentration in this crack. This proves that the distribution of bacterial carriers in the self-healing concrete was not uniform. As the time increased, the healing percentages of the cracks continued to improve, as shown in Figure 8b,c. When the healing time reached 28 days, almost all of the cracks in the SRC-L concrete were completely repaired and the maximum healed crack width was 0.86 mm. The cracks in the SRC-S specimens all reached healing percentages higher than 50%, and the maximum healed crack width was 0.52 mm. It was clear that the crack-healing capacity of the SRC-L concrete was better than that of the SRC-S concrete, indicating that the rubber particles with a larger size were more suitable for immobilizing bacterial spores in self-healing concrete. This may be due to the fact that larger rubber particles can provide more space for bacterial growth, avoiding bacteria death due to squeezing in the concrete matrix. Moreover, the bacteria can move in the cracks during the process of incubation, providing calcium carbonate crystals to those locations that do not have carriers at the beginning. This happened in Crack C in Figure 8, which had a healing percentage of zero in the first seven days after incubation, but was repaired gradually after the healing time of seven days. Bacterial transport in the cracks can help to heal cracks in locations with no bacterial carriers, improving the healing capacity of the self-healing rubber concrete.

### 3.3. The Slumps of the Self-Healing Concrete

**Figure 9:**
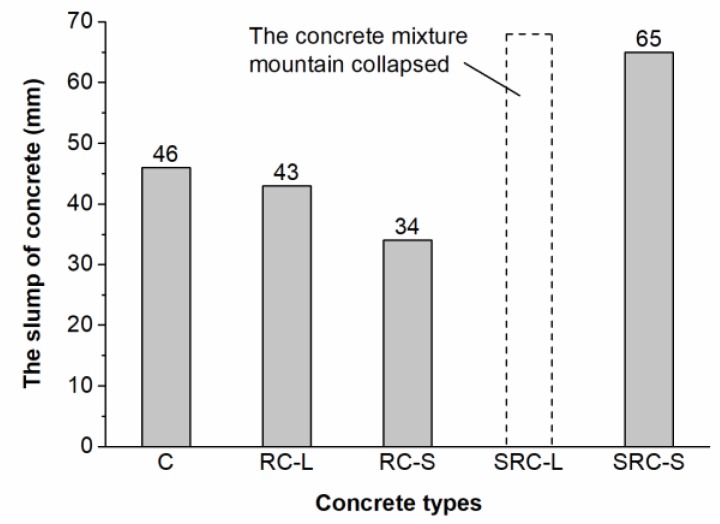
The slumps of the five types of concrete.

The slumps of the five types of concrete are shown in Figure 9. It can be seen that the slumps of the RC-L and RC-S concretes were 43 mm and 34 mm, respectively, which decreased by 6.5% and 26.1%, respectively, compared with the C concrete (46 mm). This is due to the ability of the rubber particles to absorb the free water in the fresh concrete into the pores on their surface, reducing the fluidity of the fresh mixture [21]. Moreover, the rubber particles with a small size had a better capacity to absorb water than the rubber particles with a large size, as they had a larger surface area. The SRC-S concrete had a 41.3% higher slump (65 mm) than the C concrete, whereas the fresh mixture of the SRC-L concrete collapsed when measuring the slump. These phenomena indicate that the presence of the healing agent led to a more flowable mixture. The previous studies [17,27] show that bacterial nutrients, such as yeast extract, urea, and calcium salt, which were mixed with fresh concrete in self-healing concrete, had a negative effect on cement hydration in the early age.

### 3.4. The Compressive Strength of the Self-Healing Concrete

**Figure 10:**
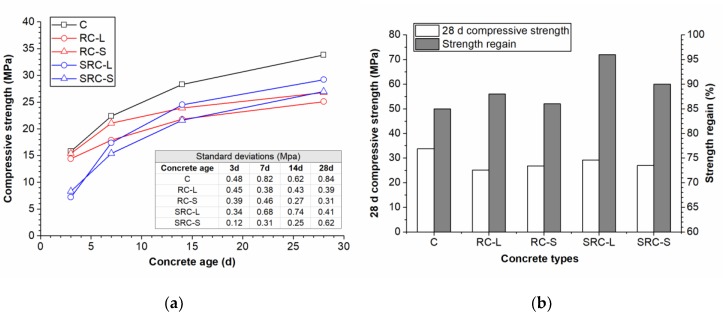
The compressive strengths (**a**) and the strength regain (**b**) of the five types of concrete.

The compressive strengths of the five types of concrete at different ages are plotted in Figure 10a. With the increase of the concrete’s age, the compressive strengths of all types of concrete improved and the rate of improvement slowed down gradually. The strengths of the RC-L and RC-S concretes at all ages were lower than those of the C concrete. At 28 days, the strengths of RC-L and RC-S reached 25.1 MPa and 26.8 MPa, respectively, which were 25.8% and 20.7% lower than the strength of the C concrete (33.8 MPa), illustrating that the rubber particles had a negative effect on the compressive strength of the concrete. This was because the rubber particles, as a soft material, can weaken the compaction of the concrete. Moreover, the rubber particles with a large size, as larger weak points, led to a further reduction in strength compared to the rubber particles with a small size. At the age of three days, the SRC-L concrete and the SRC-S concrete had compressive strengths that were lower than those of the C, RC-L, and RC-S concretes, proving that the degree of cement hydration in the self-healing concrete was low at early ages because of the negative effect of the nutrients on the cement hydration in the concrete. However, the compressive strengths of the SRC-L and SRC-S concretes exceeded those of the corresponding normal rubber concrete by 16.4% and 0.7%, respectively, reaching 29.2 MPa and 27 MPa at the age of 28 days. In the self-healing rubber concretes, some bacterial spores were activated by the free water and produced calcites within the pores of the concrete matrix [28], improving the compaction of the concrete. In addition, the larger rubber particles provided a larger space for bacterial mineralization, resulting in a greater compressive strength improvement in the SRC-L concrete. Nevertheless, the compressive strength of the SRC-L concrete at 28 days was still 13.6% lower than that of the C concrete.

Figure 10b shows the compressive strength regain of the five types of concrete. The C, RC-L, and RC-S concrete had a strength regain of 85%, 88%, and 86%, respectively, resulting from the hydration of unhydrated cement particles in the concretes without self-healing agent. The strength regains of the SRC-L and SRC-S concretes reached 96% and 90%, respectively, implying that calcites were produced in the cracks and improved the degree of repair in the self-healing concrete.

### 3.5. The Splitting Tensile Strength of the Self-Healing Concrete

**Figure 11:**
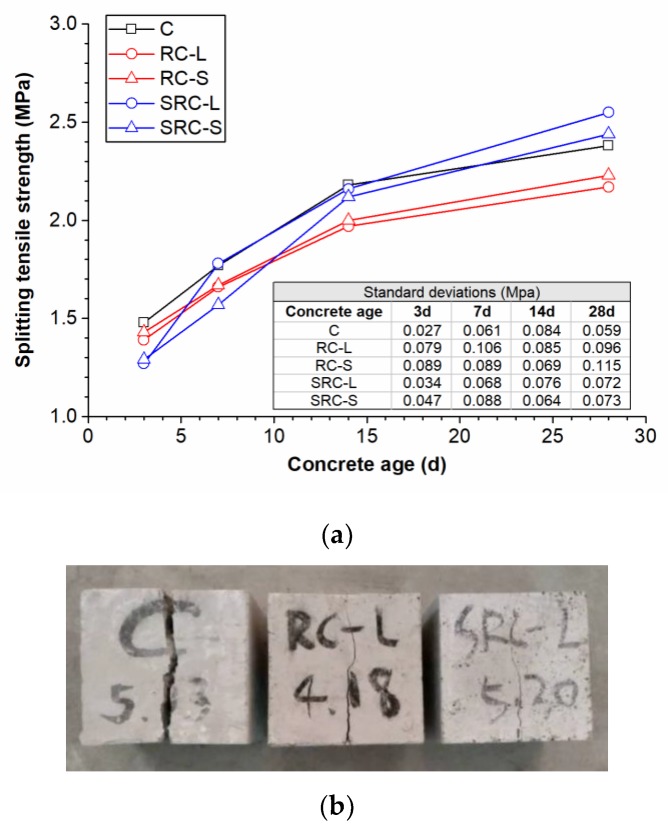
The splitting tensile strengths of the five types of concrete (**a**) and the crack characteristics of the concretes (**b**).

The splitting tensile strengths of the five types of concrete at different ages are plotted in Figure 11a. It can be seen that the RC-L and RC-S concretes had splitting tensile strengths that were lower than that of the C concrete during the curing time, and were reduced by 8.8% and 6.3%, respectively, at the age of 28 days. The reason may be that the weak bonding between the rubber particles and the cement accelerated the concrete’s breakdown [21]. The two types of self-healing concrete had low splitting tensile strengths at three days because of the low cement hydration at early ages. However, the splitting tensile strengths of the self-healing concrete began to exceed those of the corresponding normal rubber concretes from the age of 14 days. At the age of 28 days, the splitting tensile strengths of the SRC-L and SRC-S concretes were 2.55 MPa and 2.44 MPa, respectively, which is an improvement of 17.5% and 9.4%, respectively, over the corresponding normal rubber concretes (2.17 MPa and 2.23 MPa), and an improvement of 7.1% and 2.5%, respectively, over the C concrete (2.38 MPa). This may be because the SA coating on the rubber particles in the self-healing concrete can function as a coupling agent to improve the bonding of rubber particles and cement.

Figure 11b shows the crack characteristics of the C, RC-L, and SRC-L concretes after the splitting tensile strength tests. The RC-L and SRC-L concretes had more narrow cracks than the C concrete, proving that the concretes with rubber particles had lower brittleness and better anti-crack capability.

## 4. Discussion

### 4.1. The Working Mechanism of the Self-Healing Rubber Concrete

**Figure 12:**
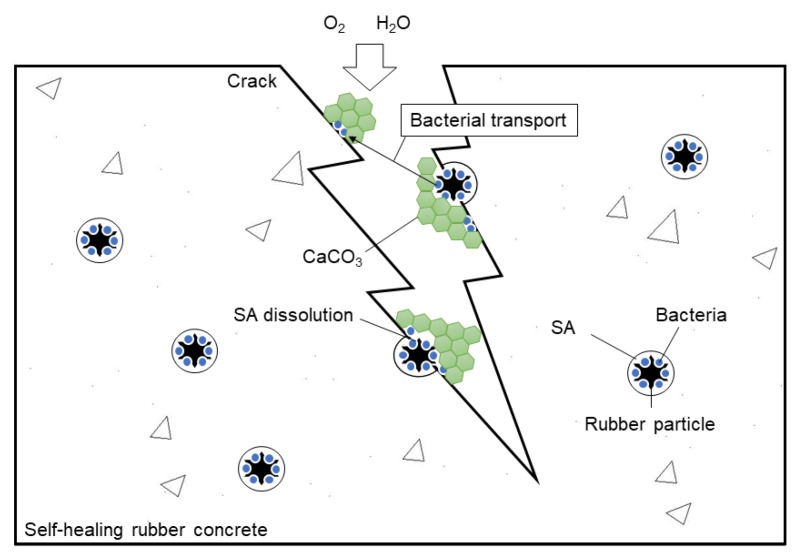
The working mechanism of the self-healing rubber concrete.

The test results have proved that the self-healing concrete using rubber particles to immobilize bacterial spores can produce precipitations in the cracks to repair them. The working mechanism of the self-healing rubber concrete is displayed in Figure 12. When the concrete was not cracked, the bacterial spores were dormant in the pores of the rubber particles, which were coated by SA. After the cracks appeared on the surface of the concrete, the oxygen and water in the external environment can enter into the concrete through the cracks. The SA, coated on the rubber particles on the surfaces of the cracks, was dissolved slowly by the water and the bacterial spores were released from the rubber particles. These bacterial spores were activated by the oxygen and water in the cracks and began to grow, using the nutrients in the concrete. Then, calcite precipitation was produced by the bacterial cells in the cracks and sealed the cracks gradually. In this process, some bacterial cells can be transported with the water flow to locations with no bacterial carriers to produce crystals in the cracks.

In the self-healing concrete, the role of the rubber particles is to:

- protect the bacterial spores from squeezing and high alkalinity in the concrete;
- provide spaces for bacterial growth and mineralization; and
- reduce the brittleness of the concrete and improving the anti-crack capability of the concrete.

The role of the SA is to:

- wrap the bacterial spores in the pores of the rubber particles;
- slowly dissolve and release the bacterial spores when cracks appear; and
- improve the bonding of rubber particles and cement.

### 4.2. Comparison of Rubber Particles with other Bacterial Carriers

A comparison between rubber particles and two other representative bacterial carriers is exhibited in Table 3. It is obvious that the method that uses rubber particles to immobilize bacterial spores has the merits of a simple procedure, savings on bacterial spores, low cost, and better anti-crack capability when compared with melamine microcapsules and expanded perlite. The bacterial spore density in the concrete of this study is reduced by an order of magnitude in comparison to the other two bacterial carriers; however, the maximum width of the cracks that can be repaired is similar to the others. This can bring about great savings on bacterial spores in practical engineering applications. In addition, the cost of the rubber particles is very cheap and the price of the bacterial carrier (rubber particles and SA) per m^3^ concrete is only 8.26 US$ (based on the price in Tianjin, China), which is lower than the price of the other two bacterial carriers. Moreover, the anti-crack capability of the concrete can be improved by the rubber particle method, as shown in Figure 11b. Therefore, the use of rubber particles to immobilize bacterial spores in self-healing concrete has the potential to be more widely used for practical engineering applications. However, it was difficult to obtain a uniform distribution of rubber particles in the concrete because their density is much lower than that of sand. This limitation needs to be further considered in future research.

## 5. Conclusions

This paper investigated the feasibility of using rubber particles as a novel bacterial carrier in self-healing concrete. Two types of self-healing concrete were prepared with different rubber particle sizes, whose healing capacities were evaluated. The properties of the self-healing rubber concrete, including the slump, the compressive strength, and the splitting tensile strength, were compared with those of plain concrete and normal rubber concrete. The following conclusions can be drawn:

- The method for immobilizing bacterial spores with rubber particles was feasible and the maximum values of the widths of completely healed cracks after 28 days of healing were 0.86 mm in the SRC-L concrete and 0.52 mm in the SRC-S concrete.
- The healing capacity of the SRC-L concrete was better than that of the SRC-S concrete because larger rubber particles can provide larger spaces for bacteria to grow and mineralize, avoiding bacteria death due to squeezing in the concrete.
- The compressive strength of the self-healing concrete was low at early ages due to the negative effect of the nutrients in the concrete on cement hydration, but exceeded the compressive strength of the corresponding normal rubber concrete at 28 days because calcites were produced within the pores of the concrete matrix.
- The splitting tensile strength of the self-healing concrete was higher than that of the plain concrete, resulting from SA improving the bonding between rubber particles and cement. In addition, the rubber particles can enhance the anti-crack capability of the concrete.
- The immobilization of bacterial spores using rubber particles has the advantages of a simple procedure, materials savings, and low cost, and provides a more suitable method for engineering applications of self-healing concrete.

Even through there are some problems with self-healing rubber concrete that need to be solved, such as the distribution of bacterial carriers in the concrete, the most suitable rubber particle size, and the protective effect of rubber particles on bacterial spores in the long term, the use of rubber particles remains an innovative and promising method for the immobilization of bacterial spores in self-healing concrete.

## Figures and Tables

**Table 1:** The bacteria carriers adopted in the published studies.

Immobilization Mode	Bacteria Carrier	Immobilization Method	Maximum Crack Width (mm)
Encapsulated	Epoxy [17]	Making microcapsules; Encapsulating bacterial spores and other healing agents; Filling the protection materials into the microcapsules.	0.3
	Poly [15]		0.4
	Melamine [16]		0.97
	Hydrogel [18]	Mixing bacterial spores with polymer solution and an initiator; Degassing and subjecting to UV irradiation. Freezing and drying.	0.5
Adsorbed	Diatomaceous earth [19]	Impregnating the carriers with a bacterial spore suspension; Drying in an oven; Coating with protection materials.	0.17
	Expanded perlite [7]		0.79
	Expanded clay [1]		0.46
	Ceramsite [20]		0.51

**Table 2:** Mixing proportions of the concrete specimens.

Concrete Type	C	RC-L	RC-S	SRC-L	SRC-S
Cement (kg/m^3^)	350	350	350	350	350
Sand ^1^ (kg/m^3^)	750	675	675	675	675
Coarse aggregate (kg/m^3^)	1110	1110	1110	1110	1110
Water (kg/m^3^)	190	190	190	190	190
Yeast extract (kg/m^3^)				1.4	1.4
Ammonium sulfate (kg/m^3^)				0.7	0.7
Calcium acetate (kg/m^3^)				7	7
Urea (kg/m^3^)				7	7
Rubber particles ^2^ (kg/m^3^)		33	33	33	33
Bacterial spores (pores/m^3^)				3.75 × 10^13^	3.75 × 10^13^

^1^ apparent density of 2547 kg/m^3^; ^2^ apparent density of 1120 kg/m^3^.

**Table 3:** Comparison of the rubber particles to other bacterial carriers.

Bacteria Carrier	Melamine Microcapsule [16]	Expanded Perlite [7]	Rubber Particle
Immobilization procedure	A patented polycondensation reaction-based microencapsulation process; Melamine-based, and contains an inert substance to protect the spores;	Impregnating the expanded perlite particles under vacuum with the bacterial spore suspension; Drying the particles in an oven; Spraying the nutrients onto the surface of the particles; Coating the particles with a geopolymer coating	Mixing the rubber particles, the SA solution, and bacterial spore suspension together; Stirring them adequately; Drying in a cool and ventilated place.
Bacterial spore density	1.8 × 10^14^ cells/m^3^ concrete	5 × 10^14^ cells/m^3^ concrete	3.75 × 10^13^ cells/m^3^ concrete
Maximum crack width	0.97 mm	0.79 mm	0.86 mm
Price	Expensive	9.18 US$/m^3^ concrete	8.26 US$/m^3^ concrete
Other characteristics			Higher anti-crack capability
